# Metasynthesis: Experiences of Women with Severe Maternal Morbidity and Their Perception of the Quality of Health Care

**DOI:** 10.1371/journal.pone.0130452

**Published:** 2015-07-01

**Authors:** Mohd Noor Norhayati, Sukeri Surianti, Nik Hussain Nik Hazlina

**Affiliations:** 1 Department of Family Medicine, Universiti Sains Malaysia, Kota Bharu, Kelantan, Malaysia; 2 Department of Community Medicine, Universiti Sains Malaysia, Kota Bharu, Kelantan, Malaysia; 3 Women Health Development Unit, Universiti Sains Malaysia, Kota Bharu, Kelantan, Malaysia; Harvard Medical School, UNITED STATES

## Abstract

**Aim:**

To explore the experiences of women with severe maternal morbidity and their perception of the quality of health care.

**Background:**

The exploration of factors associated with severe maternal morbidity has emerged as an alternative strategy in reducing maternal mortality. This approach is useful for the evaluation and improvement of maternal health services.

**Design:**

Included a comprehensive search, appraisal of reports of qualitative studies, the classification of studies and the synthesis of findings.

**Data Sources:**

A literature search was conducted through nine databases for articles published between January 1980 and August 2013.

**Review Methods:**

The quality of included studies was assessed with a modified Critical Appraisal Skills Program tool. The synthesis applied a meta-ethnographic approach. It involved (1) identifying and comparing the findings; (2) creating a parsimonious thematic structure and (3) searching for disconfirming data.

**Results:**

Nine studies published between 2005 and 2012, involving 292 women with severe maternal morbidity, were included. Three key themes were identified: 'provision of care', 'severe maternal morbidity' and 'health care seeking behavior'. Barriers to the access and utilization of heath care services were identified.

**Conclusion:**

The findings appear to suggest that mental and physical health outcomes of women who experienced severe maternal morbidity were poor. There is a need to identify the persistence and severity of these outcomes over a longer period of time. More realistic and less biased information may be obtained in community-based interviews. The impact of potential negative fetal outcomes would be a strong influencing factor for the women. These findings may help to increase awareness of the non-physical components of severe maternal morbidity and provide guidance for professionals regarding preventive measures.

## Introduction

One of the Millennium Development Goals (MDGs) is the prevention of the avoidable loss of women’s lives in pregnancy and childbirth. The target of this MDG is a 75% reduction in maternal mortality worldwide by 2015. Nevertheless, over 270,000 women still die from pregnancy-related causes annually around the world, and a further 9.5 million women are estimated to suffer from pregnancy-related complications [1].

Very severe life threatening and severe potentially life-threatening maternal morbidity that occur during pregnancy, childbirth or after termination of a pregnancy [2] have emerged as a complement to confidential maternal-death enquiries or as an alternative strategy to reduce maternal mortality [3, 4]. Maternal morbidity has been viewed as a useful outcome measure for the evaluation and improvement of maternal health services in developing countries [3] and is regarded as providing superior information on the pathways that lead to severe morbidity and death, in terms of the burden of disease, quality of care [5] and rapid reporting on maternal care issues [3, 6], because the women survive.

These women who survive severe obstetric complications comprise a vulnerable population who often suffer from both the immediate and longer-term physical, social, financial and psychological consequences of severe maternal morbidity [7]. They can provide insights into risk factors as well as potential strategies for prevention of maternal morbidity and maternal mortality, as they share similar characteristics with the women who have died due to obstetric complications [3, 6].

Metasynthesis is a method that brings together qualitative exploratory studies to enhance their contribution to the development of more formalized knowledge [8]. It seeks diversity in studies to explore how disparate findings are conceptually related to one another and to clarify the defining and overlapping attributes [9]. Findings from qualitative studies have important implications for knowledge development, and for these findings to have an impact, they must be situated in a larger interpretive context and presented in an accessible and usable form [10]. This review is important for furthering understanding about how the findings are conceptually related to one another and for clarifying the attributes [9], thereby providing a global picture of the phenomenon [11]. Hence, the aim of this metasynthesis is to explore the experiences of women with severe maternal morbidity and their perceptions toward the quality of health care.

### Theoretical Framework

The synthesis was guided by the quality of care framework for severe maternal morbidity [12], Andersen behavioral model [13], as well as by findings from articles and team discussions. The quality of care framework for severe maternal morbidity depicts the experience of care received and the experiences of women with severe maternal morbidity. The quality of care received comprised the evaluation of factors such as the provider-client information (communicating the situation and the outcomes to the patient), interpersonal relations (including the attitude of the providers) and human and physical resources. The factors that women discussed in relating their experience of severe maternal morbidity included coping mechanisms, the impact of losing a baby, thoughts and perceptions of death and how the women perceived the care they received, which in turn influenced their satisfaction [12].

The Anderson model explains the health care seeking behavior of individuals, which was categorized into an individual's predisposition to use medical services, enabling or impeding circumstances and the need for health care. Predisposing characteristics related to demographic elements and social structures included age, gender, residence, occupation, education, ethnicity and attitudes regarding one’s health. Enabling elements consisted of community factors that affect the availability and accessibility of health care; and personal factors such as knowing how to take advantage of what is offered. Finally, the needs for health care included the type of illness, perceived health status and the expected treatment outcome [13].

After providing a brief overview on the narratives of women in this review, we describe the experiences of women who experienced severe maternal morbidity and the quality of health care received, organized into the following themes: perception of the provision of care, severe maternal morbidity experience and health care seeking behavior. These elements are interrelated and influence how the women cope with severe maternal morbidity and their perceptions regarding the quality of care.

## Method

The research was approved by the Human Research Ethics Committee (HREC), Universiti Sains Malaysia (FWA Reg. No: 00007718; IRB Reg. No: 00004494) and Medical Research Ethics Committee (MREC), Ministry of Health (KKM/NIHSEC/800-2/2/2/Jld 2 P13-215).

### Design

The design of this review included (a) a comprehensive search, (b) appraisal of reports of qualitative studies, (c) classification of studies, and (d) synthesis of the findings. The meta-ethnographic approach [10] was applied to generate the findings or theoretical insights in exploring the experience of women with severe maternal morbidity. This approach can be applied to a full range of qualitative methods and is not limited to only ethnographic studies. Several findings from one particular paper were identified and compared with the findings from another paper to generate the initial concepts. Similar concepts were then collapsed to create a parsimonious thematic structure in a process termed 'reciprocal translation'. Each author then reviewed the themes independently to ensure that no data were left unexplained or explained differently. This process of searching for disconfirming data is termed 'refutational translation'. The themes were then synthesized into a 'line of argument', which is a phrase or statement that summarizes the main findings of the study.

### Search Methods

The search methods included (a) a review of studies that examined the experience of women with severe maternal morbidity (including very severe maternal morbidity), and (b) qualitative and/or mixed methods designs. Studies were limited to (a) those published or available in English, (b) studies in peer-reviewed journals, and (c) those undertaken between January 1980 and August 2013. The literature search was conducted using the following databases: MEDLINE, CINAHL, the Cochrane Library, PubMed, PsycINFO, Scopus, Science Citation Index, EMBASE and BMC. Search terms included the following: near miss morbidity, near miss, severe maternal morbidity, severe acute maternal morbidity, obstetric near miss, maternal near miss and obstetric near miss.

### Search Outcome

In total, 276 citations were retrieved, and after reviewing the title and abstract, 265 articles were excluded because they failed to address the initial selection criteria (S1 Fig and S1 PRISMA Checklist). The studies were either quantitative studies or were unrelated to the experience of severe maternal morbidity. The full articles of the remaining 11 citations were obtained and reviewed. Two articles were excluded as they were not published in English; ultimately, nine articles were taken forward for quality appraisal.

### Quality Appraisal

The quality of each study included in the review was assessed with a modified version of the Critical Appraisal Skills Programme (CASP) tool [14]. The CASP provides a standardized mechanism for appraising qualitative studies [15]. It consists of 10 questions covering the credibility and relevance of the studies and has been used in previous reviews of qualitative studies [16–18].

The first two questions screened for relevance and appropriateness of the studies and the following eight questions assessed the research design, sampling, data collection and analysis, reflexivity (research partnership relations / recognition of researcher bias), ethics, findings and the value of the research. A scoring system applied by Duggleby *et al*. (based on a three-point rating system developed by Feder *et al*.) was used in this review [15, 18]. For each question, a weak score (one point) was assigned to articles that offered little to no justification or explanation for a particular issue (e.g., where, when, or how the data were collected was not mentioned). A moderate score (two points) was given to articles that addressed the issue but did not fully elaborate on it (e.g., the justification for using constant comparisons was presented but the procedure itself was not explained). A strong score (three points) was assigned to articles that extensively justified and explained the issue at hand (e.g., the authors explained that semi-structured interviews were used, transcribed verbatim and modified part way through the study, and then offered some sample interview questions). For each article, a score was calculated for all eight questions and then totaled, with a possible maximum score of 24. For the nine articles included, the CASP score ranged from 11 to 24 with the mean (SD) score being 17.1 (3.66).

### Data abstraction and synthesis

Table 1 presents a summary of included studies and their purpose, methods and quality appraisals. The stated methodological approaches employed were: (a) thematic analysis [12, 19–25] and (b) discourse analysis [26]. The findings from the studies were managed using NVIVO 10 software. It is software that allows users to classify, sort and arrange information by coding and generating the list of code categories. The code categories formed were based on the analytic expertise of the users. Code categories were refined as subsequent data were gathered. The initial concepts identified the experiences of women with severe maternal morbidity and their perceptions toward the quality of health care as reported in the studies. Following the interpretations or conclusions of the primary studies, emerging themes were formed. Interpretations or conclusions of the metasynthesis formed the final themes. The constructs were also assessed in relation to the quality of the included studies.

**Table 1:** Summary of articles in the metasynthesis.

Source and country	Purpose	Method	Finding classification and CASP total
Carvalheir *et al*. (2010), Brazil	To understand severe maternal morbidity from the perspective of women who experienced it.	Interview with 16 women at the hospital after discharge	Collective Subjective Discourse,CASP = 11
Jonkers *et al*. (2011), Netherland	To study patients' perspectives on ethnicity-related factors contributing to sub-standard maternity care and to explore the possible relationship between sub- standard care and severe maternal morbidity among immigrant women.	Interview with 40 immigrant and 10 native Dutch women. 46 of the interviews were conducted in the homes of the interviewees between two and six weeks after discharge from hospital, and 4 in hospital because of their relatively long hospitalisation.	Thematic, CASP = 14
Roost *et al*. (2009), Bolivia	To identify the social, familial, economic, knowledge, and empowerment factors in the healthcare seeking process and the major barriers perceived by women in accessing appropriate care.	Semi-structured in-depth interview with 30 women with a near-miss event upon arrival at hospital.	Thematic, CASP = 15
Sikder *et al*. (2011), Bangladesh	To describe the health care decision-making process during severe acute obstetric complications among women and their families.	Semi-structured, in-depth interviews with 40 women reporting severe acute obstetric complications at 1-month postpartum	Thematic, CASP = 17
Souza *et al*. (2009), Brazil	To investigate the emotional experiences of women who survived severe complications related to pregnancy.	Semi- structured interviews with 30 women on the 5th day postpartum before discharge.	Thematic, CASP = 18
Storeng *et al*. (2008), Burkina Faso, South Africa	To compare the experiences of women who experienced life- threatening obstetric complications with women who delivered without complications.	Structured in-depth interview with 82 women (18 women with uncomplicated delivery and 64 women with near-miss at 1 week to 1 month and 6 months postpartum. Only 13 were also interviewed at 12 months).	Interpretive and thematic, CASP = 17
Storeng *et al*. (2010), Burkina Faso, South Africa	To compare the experiences of women who had experienced ‘near-miss’ events during pregnancy and childbirth with women who had ‘uncomplicated’ deliveries.	Structured in-depth interview with 82 women (18 women with uncomplicated delivery and 64 women with near-miss at 1 week to 1 month and 6 months postpartum. Only 13 were also interviewed at 12 months	Interpretive and thematic, CASP = 19
Tuncalp *et al*. (2012), Ghana	To explore women’s experiences of severe maternal morbidity and perceptions of the care they received	Semi-structured interview with 32 women prior to hospital discharge.	Thematic, CASP = 24
Weeks *et al*. (2005), Uganda	To record the experiences of women who with ‘near-miss’ maternal mortality.	Semi- structured interview with 30 women during hospitalization.	Thematic, CASP = 19

### Validity

The validity of this metasynthesis was maintained by (a) a comprehensive literature search, (b) team discussions and decisions on search terms and inclusion criteria, and (c) an assessment of the appraisal and assignment of CASP scores. Two reviewers (NMN and SS) independently appraised each study, and presence of any differences was resolved by discussion or adjudication by a third reviewer (NHNH). The team also discussed the findings of the studies and themes until agreement was reached through consensus.

## Results

The quality of the nine studies was summarized using the CASP tool, based on a three-point rating system for eight questions covering credibility and relevance of the studies. The resulting scores showed the ranking of the methodological quality of the different studies. Nine studies showed moderate to good quality with adequate justification and explanation [12, 19, 20, 22, 23, 25]. While the quality was generally good, three studies showed weak to moderate quality with limited elaboration of issues [21, 24, 26].

The nine studies included in the metasynthesis represent the views of women from seven countries (Table 1), comprising 292 women with severe maternal morbidity and 18 women with uncomplicated deliveries for comparison. Storeng *et al*. produced two different papers on the cost, social and economic consequences [20] and consequences with regard to health and well-being [23] of the same group of women. The earliest paper was published in 2005 and the most recent in 2012, with the majority (*n* = 7) being published within the last three years, suggesting an upswing in interest in this area of research. Five of the studies included interviews that were conducted upon arrival to the hospital [21], during hospitalization [12, 19, 22] and after discharge [26]. Four of the studies included interviews that were conducted in the community between one and six weeks postpartum [20, 23–25] and a follow-up interview was conducted at six months [20, 23]. Two studies published by Storeng *et al*. were conducted on a similar cohort of women but on different factors [20, 23].

By synthesizing the initial concepts and emerging themes, we identified three final themes in the experience among the women with severe maternal morbidity (Table 2). These themes represent our interpretation, across the studies, about what the women felt.

**Table 2:** Summary of concepts and themes.

Initial concepts	Emerging themes	Final themes
Timing of assessment/evaluation of patient	Quality of medical	Perception on
Appropriateness of diagnosis	care	provision of
Timely diagnosis /recognition of high-risk status		care
Timely referral		
Timely treatment		
Appropriateness of treatment		
Contact time with qualified staff	Provider-client	
Necessary medical information	information and	
Prepared for treatment and understand their options	relationship	
Reason for specific information were clearly explained		
General attitude of health staffs		
Competent staff	Human and	
Inadequate number of staffs	physical resources	
Physical infrastructure and overall environment	within health setting	
Limitations in physical capability or physical changes	Physical experience	Severe maternal
Dealing with memory gap		Morbidity experience
Thoughts and perception of death	Emotional experience	
Distress		
Fear		
Discouragement / hopelessness		
Scared of operation or hospital		
Feeling of blame		
Frustration		
Isolation		
Displacement of emotion	Coping mechanism	
Religious faith		
Considering next pregnancy		
Giving more values to life		
Abandoning risky behaviors		
Dealing with loss		
Attitude towards health	Women's	Health care
Woman powerlessness	predisposition to use	seeking
Perception of health care	medical services	behavior
Cultural practice and beliefs		
Medical cost and funding		
Language barrier	Enabling or impeding	
Transportation	circumstances	
Modern mode of communication		
Recognition of seriousness of condition	Need for health care	

### Theme One: Perception Regarding Provision of Care

This theme incorporates three emerging themes, which include the quality of medical care, provider-client information and relationship, and human and physical resources within the health care setting.

#### Quality of medical care

Women in most of the studies were unsatisfied with the health care services because of the delay and inappropriateness in the management of patients. The types of delays included a delay in assessment or evaluation of patients [12, 24], and a delay in diagnosis [24], referral [19, 24] and treatment [19].

‘I was laid down for many hours without a doctor attending to me. Later on, a nurse came to me and examined me and she called a doctor. Then, the doctor came in and asked me to go and do a scan. I stayed there for a long time even before I was taken to the delivery ward for the scan. I went to the delivery ward I was just lying down for so many hours. It was around 3:30 am that another doctor found out that I’ve been there and I was bleeding then and later they said it’s too late for me…'[12].

For some women, the problems involved inappropriate diagnosis and management. One patient presented with vaginal bleeding and was thought to have a pelvic infection but was found to have an ectopic pregnancy. Another patient had a pelvic infection as a consequence of untreated retained placenta [19]. In one case, contractions in a woman at 36-weeks gestation were mistaken for Braxton Hicks contractions. The unexpected birth of the baby caused the midwife to panic, and she pulled the placenta too forcefully, leading to a major obstetric hemorrhage [24].

#### Provider-client information and relationship

Some women complained about the ways in which health care providers treated them. These behaviors included a lack of contact time, not providing necessary information in an understandable language and an inhumane attitude.

'Sometimes there are few of them for many of us, but it is better to spend a longer time with the patient and provide a better quality of care than to do everything quickly and sometimes complicate the person’s situation'[22].

'If someone had told me what was going on, I would have felt okay… The most important thing about that obstetrician was that he had not told us everything in detail' [24].

'The staff is harsh. They don't listen to you and they don't explain anything that they're doing. You just lie alone in your bed feeling scared and lonely. You don't know why you can't go home or what they are doing to you' [21].

Although some women felt traumatized by their experience with health care providers, expressions of thanks were not uncommon.

'The doctor is very good, the one who took me to the theatre. Because he told me everything, and everything he did, he wanted you to know. He would tell you that I am doing this and that and that etc., so I always say that I am happy with that' [12].

#### Human and physical resources within the health care setting

Seventy percent of women reported being taken to certified health care providers because their situation had become desperate and the non-certified providers were unable to handle the complications. However, the general impressions of government facilities were that they were crowded and that there was a shortage of qualified doctors [25]. Some women also mentioned the lack of physical resources such as water, mosquito nets and delivery beds [12]. However, some women accepted the limited resources and appreciated the work conducted within those limits. One woman summarized the situation by saying,

‘The beds are few but the patients are very many’ [19].

### Theme Two: Severe Maternal Morbidity Experience

#### Physical experience

Physical condition during this critical state of health was an important factor for the women. In general, the occurrence of a severe complication during the pregnancy and delivery was an unexpected event. Such an occurrence was associated with unpleasant sensations and discomfort due to the condition and its treatment [22, 23].

'And all that afternoon, I was taking (magnesium) sulphate and feeling bad just the same. Feeling my heart beating and the burning sensation… I thought the feeling of death must be just like this, really' [22].

Some of the women perceived that the abnormalities might be long lasting and have possible social implications, especially untoward reactions from their spouses and the unfulfilled responsibilities of raising their children while the women were incapacitated [22]. Some women reported that not knowing what had taken place, due to their altered level of consciousness or remaining sedated for long periods of time because of the mechanical ventilation, was the most difficult part of their delivery experience. Awakening to the discovery that significant events such as a hysterectomy or the death of a baby had already occurred might be very traumatic. When the women did not remember aspects of the events that occurred, it made them upset and distressed, and they found it more difficult to accept the losses in such cases [12, 22].

#### Emotional experience

Many women felt the sensation of the transitory nature of human life. They felt that life was short and death was imminent, and in some cases, they thought that they were already dead. Such experiences led to concerns about loved ones, particularly their children [12, 22]. The perception that death was close was striking and present throughout the entire experience of women with severe maternal morbidity.

'I don’t even know how to say it. I have not seen such a thing before in my whole life. I have suffered a lot. I thought I was dead. I cried continuously for three days… I was thinking I was going to die. At the theatre, I thought all was over. I was crying and begging… I cried because I was thinking I was going to die and leave my two kids behind… The thing that was happening to me, I knew I was on the path of death' [12].

Negative forms of emotion such as fear, frustration, unhappiness, hopelessness, and feelings of blame and isolation were reported. Fear appeared to be the driving force underlying the sensation of impending death. The fear could be mild or terrifying and paralyzing [22, 26]. The consequences of Cesarean sections [12, 21, 25] and transfers to hospital and the intensive care unit [22] were worrisome and made the women feel that their condition was quite severe.

The occurrence of an unexpected complication that led to the loss of an otherwise normal pregnancy was met with frustration by some women. It was as if they were incapable of performing the physiological process of reproduction [12, 22]. The experience was also described as painful when it resulted in the birth of a child who was unhealthy [22, 26]. However, severe maternal morbidity was also viewed as something that could end well when appropriate care led to the newborn’s improvement and its subsequent inclusion in the family environment [26].

Latent feelings of blame may appear during a severe complication. Some women viewed the complications as a form of punishment or as a consequence of their own behavior [19, 22, 26]. The inability to identify past behavior led them to feel that the complication was unfair [22].

'After operating on me, they came and told me that they removed my uterus, which means I will never produce a baby…. It was my mistake. I aborted so I could not say anything, because I allowed it' [19].

'I am suffering for an error that I did not commit… I am sure I did nothing' [22].

For many women, severe maternal morbidity was accompanied by great emotional distress; thus, support from the people closest to them (such as the husband or family members) was extremely important [21, 22]. However, a form of isolation was also experienced, given the difficulty of women in relating to other people during the complication. This difficulty hindered the sharing of suffering, even with those closest to them. Nevertheless, some women felt that they themselves or their families had difficulty talking about this difficult time [22].

#### Coping mechanisms

Religious faith has been reported in many studies as one of the main coping mechanisms. In facing these critical situations, many women attributed the solution to their problems to God and accepted the loss as being God’s will. Once they experienced the situation, they expressed their relief at having the situation under control and joy at being alive. Religious faith resulted in hope and strength, which enabled the women to go on, to fight and to cope with difficulties. This attitude is considered to be integral to the recovery process and as such brought them comfort [12, 22, 25, 26].

Some women found something positive in the experience and viewed life in a different way. They expressed the importance of placing more value on their family and the people who were truly fond of them. Additionally, they placed more value in the simple things in life and less value in material things [22].

'I think that in one way it was good… I think I was needing to be shaken up… Because sometimes we give importance to people who aren’t worth it… to things that don’t deserve it… I think I really had to go through this' [22].

Another important aspect of the experience of severe maternal morbidity is the displacement of emotions from the woman to the child. Even when the risk of maternal death was real, many women focused their attention not on their own health but on the well being of the baby or on their other children [22, 26].

I was afraid of dying because of my children…. I have a little one 4 years old and now I have the baby; that’s why I was afraid of dying' [22].

Loss of the baby influenced the experience of the women and their coping mechanisms. Women with a live baby reported feeling happy at the end of the event, while women who lost their babies reported lingering feelings of sadness, grief and concern regarding the future of their marriage [12, 22]. The birth of a preterm baby is often associated with the risk of sickness and death. Mixed feelings of guilt, anxiety, concern and confusion in the parents, coupled with early separation due to the transfer of the baby to the neonatal unit, led to feelings of loss and early mourning [26]. Some women coped with this significant loss by considering a future pregnancy or even abandoning risky behaviors such as smoking or by focusing on control of their blood pressure [22].

### Theme Three: Health Care Seeking Behavior

#### Women's predisposition to use medical services

Attitudes towards one’s health, such as delaying the decision to seek appropriate care, is a significant issue. Some women admitted to delaying the seeking of help [21, 22] until they or a close relative perceived their symptoms as being directly life-threatening. The delay was common in cases involving non-certified health care providers [25] and home birth [21]. The familiarity and proximity of these providers, flexibility in payment schemes and the perception that the illness was non-medical in origin (such as being due to evil spirits), were the reasons for their decision to delay care [19, 25].

Women who opted for home deliveries seemed to be disconnected from the health-care system. They stated that hospitals were 'not for them,' saw themselves as 'outsiders' and had vague notions about hospital delivery. Women who favored home birth perceived hospitals as being dangerous places where one had an increased risk of serious complications. The consequences of Cesarean sections were perceived as risky and possibly life-threatening. The women were worried that the operation performed was for reasons they did not understand and would interfere with their ability to work in the future. Some had heard that women were not well treated in hospitals and were not informed about their condition [21]. Some women wanted to maintain their privacy and avoid gossip when they left their homes to seek health care [25].

Women’s descriptions of their powerlessness were reflected in all aspects of their lives. Family members, particularly the husbands, retained a prominent role in the women’s actions with respect to health care decisions. Generally, the women will first inform the female family members of any severe obstetric complications, and these family members will then advise them to remain silent and endure the pain because verbally expressing the pain is considered to be undisciplined [25]. The women waited to inform their husbands or other male relatives (such as fathers, fathers-in-law and uncles) until they could no longer endure the pain. However, many women felt that their husbands would still want them to stay at home to give birth and would delay seeking medical treatment from a certified provider [19, 22, 25].

'My husband will force me to stay in our home. No matter if I live or die, I must stay at home' [25].

Cultural practices such as polygamy may also limit women’s decision-making power [25]. Often, the women’s husbands did not have enough money and did not pay enough attention to their family’s needs, including health care needs [19, 25]. Cultural beliefs that women were 'polluted' and unclean after childbirth were also responsible for the presence of traditional birth attendants or non-certified health care providers during and after delivery. Attendants’ tasks were not limited to assistance during delivery but also included helping with housework after the women gave birth [25].

Women were also under pressure to balance medical advice and social pressures. Those who had undergone invasive surgery were advised, for physiological reasons, to wait two to three years before attempting another pregnancy. However, some women feared that delays in achieving a successful pregnancy would weaken their relationship with their husband and were in tension with the social imperative for married women to achieve another pregnancy quickly. The pressure for childbearing was very strong, despite the fact that another pregnancy could be perilous to their immediate health and survival [23].

Financial deprivation was an important personal impeding factor [19–21, 23–25]. Although maternity care is provided for free in government hospitals, the women were worried about hidden costs for medicine and other items [12, 19, 25]. Cost is not a major obstacle in seeking emergency health care, but it is a significant factor in non-emergency situations. This situation influenced their decisions about having regular antenatal check-ups or arranging for hospital delivery [21].

#### Enabling or impeding circumstances

Immigrant women facing language barriers were unable to clearly convey their complaints or to understand information given about their diagnosis and treatment. Translators did not always translate information and their complaints well. Some women were aware of the negative consequences of language barriers to communication because no one other than themselves could present their complaints accurately [24].

Various means of transportation were used to reach the hospital when complications occurred. Some women used their own transportation, and others used public service ambulances. Some women did not emphasize this factor as a cause of any additional delays or problems [19, 22]. However, the journey to reach the health facility was difficult due to bumpy roads or long distances, or travel requiring the use of multiple forms of transportation such as ambulances, buses and rickshaw vans [19, 25]. The use of mobile phones by male relatives to coordinate logistics has helped the women to reach emergency care in time [25].

#### Need for health care

Some women had a lack of knowledge about the signs during a pregnancy that could indicate possible complications. Some thought that they were having a normal experience during their pregnancy and were uncertain about the significance of the signs or felt that they were not severe enough to seek immediate medical attention. They preferred to ignore the signs and wait for the symptoms to pass. Female relatives were seldom consulted about these health problems [21, 24].

'I had a headache, strange sounds in my ears, and I saw lights in front of my eyes. But, I didn't think that it could be dangerous' [21].

## Discussion

This metasynthesis explores the experience of women with severe maternal morbidity. The quality of the studies included in the review was generally good, therefore, the results were valid and acceptable.

### Theme One: Perception on Provision of Care

An important aspect that was revealed and needs to be considered regarding the pregnancy, delivery and puerperium periods is the quality of medical care and provider-client relationship. The patients' evaluation of the occurrences in severe maternal morbidity cases may or may not be the same as that of the providers. As such, this metasynthesis provides additional elements in the evaluation of the quality of maternity care [24]. Delays that include the woman or her family waiting to seek care, delays in reaching appropriate health care facilities and delays in receiving adequate care have long been recognized in overall patient management and were frequently observed in previous studies [12, 19, 24, 27]. In these studies, the documented reasons for the delays included the requirement to disinfect the operating theater following an infective surgery and the lack of theater space and staff [19].

Inadequate or inappropriate diagnosis or recognition of high risk and inappropriate treatment and inadequate documentation were the most common preventable events. This situation represents a potential causal chain where poor diagnosis may lead to inappropriate, inadequate or even the absence of treatment. Additionally, inadequate charting may reflect indecision in both diagnosis and treatment options [28]. Based on a 5-factor scoring system for classification of maternal morbidity, women with near-miss morbidity were four times more likely to have these provider-related preventability factors, compared to women with other severe but non-life-threatening morbidity. This finding indicates that changes in health care providers' behavior may have a significant impact on the women [29].

Women's perception of good quality of care includes adequate provision of information, good communication and the attitude of health staff. Women require information about their condition and treatment protocols and need to be given the opportunity to ask questions and receive clear answers. Although in certain circumstances this was not possible as the women were unconscious, it is important to highlight the fact that having access to such information calms their worries and makes them feel safer. Positive doctor-patient interactions may also improve their coping mechanisms and self-confidence, leading to improved future health care seeking [12, 24]. Gratitude for the care received may reflect either optimal care or, on the contrary, the women's low expectations [19].

### Theme Two: Severe Maternal Morbidity Experience

The occurrence of disagreeable physical experiences triggered by a severe pregnancy complication leads to the feeling of impending death and fear. Women may then be involved in a complex process of reviewing their life history and future expectations. Those dealing with loss or sick babies were at risk of experiencing adverse psychological consequences [12, 22, 26]. This situation has been described as 'maternal near-miss syndrome' [22].

'Failure' of the normal pregnancy damages the woman’s self-perception and hampers the fulfillment of some of her social roles [22]. Appropriate doctor-patient interactions involving the provision of adequate information and care for both physical and emotional aspects should be ensured for the mothers. Considering maternal needs, respecting their expectations and individuality, and including them in the child’s recovery, could provide more security, higher self-esteem and trust for the women [26].

The loss of a baby negatively influenced the women's perspectives and coping mechanisms. They were more likely to have poor mental health by three months postpartum compared to women who did not have a complicated delivery [7]. Due to the vulnerability of this group towards postpartum morbidity, they require extra attention, including counseling and postpartum visits from their health care providers [7, 22, 30]. The women often anchored themselves in religious beliefs because they believed that God would give them strength in overcoming their problems [12, 26]. Therefore, it is important for health care providers to acknowledge the religious needs of the women and include this as a strategy to increase women’s ability to overcome difficulties [26].

### Theme Three: Health Care Seeking Behavior

Many women criticized health care providers for being inattentive to them, suggesting the occurrence of substandard care. However, a delay in the decision to seek help was also observed [21, 22, 25]. The extent to which the delays were either due to lack of recognition of the seriousness of the condition, difficulties in accessing care or difficulties in undertaking natural roles, remains unclear [22].

Some women understood and recognized the severity of their condition but were unable to translate this knowledge into the prompt action of seeking health care because these decisions were made by others [25]. In this regard, interventions to improve the timely seeking of medical care that are targeted to husbands and family members are needed. Nevertheless, lack of decision-making power due to early age at marriage and low maternal education would require societal improvements in the women's status [7, 31]. Women’s empowerment is essential in health promotion programs if maternal mortality and morbidity are to be reduced [19].

The perceptions of being mistreated in the hospital and deprived of essential information led to a lack of confidence and distrust in the health care system. These factors strongly influence the utilization of maternity care services [21, 32, 33]. Distrust in health care providers leads to fear of Cesarean sections, thus causing women to avoid utilization of the delivery care [34]. Maternal care-seeking behavior is also shaped by social differentiation. The perception of being separated from other social categories due to socially demographic disadvantages suggests that the seeking of care is a socially structured practice rather than a reflective decision-making process carried out by the women [21].

Women’s powerlessness was observed, as they generally do not participate in decision-making and are only informed about any medical decisions that are made. They were led to think and act according to the convenience of the health care system [26]. However, it is important to note that the involvement of women depends on the nature of care, whether it is emergency or elective, acute or chronic, or if the women have the motivation or ability to become involved [24].

Care seeking from non-certified health care providers is still prevalent in certain developing countries. Cultural preferences for traditional birth attendants or village doctors were thought to have a role in preventing or delaying women from seeking health care. However, one study reported that this situation was more likely due to social exclusion related to ethnic background [21]. Give the lack of knowledge and skills among non-certified health care providers [19], training them in identifying serious complications and helping to refer the women to health care centers would greatly assist the health care system.

Cost was another important issue. Barriers related to distance and transportation may impede access to maternal health care, especially for poor women [21]. In the event of severe maternal morbidity, the hospital stay tends to be longer and, thus, the hospital charges escalate rapidly. Other expenses (such as drugs that are not available in the hospital pharmacy) may further add to the total cost and cause distress to the women and their families [12].

Language barriers may be overcome by the presence of professional interpreters. However, focused questioning of complaints (which may be vague) and attention to individual circumstances may help in the early diagnosis of maternal morbidity. Thus, competent diagnosis and decisive action by health care providers are more important than patient-centered participation [24].

A major strength of this review is that it explores the experience and perceptions of women who survived severe obstetric complications. It provides insight into the risk factors and potential strategies for the prevention of maternal morbidity and mortality as they share similar characteristics with women who have died. We identified qualitative studies that were completed worldwide, and most of the literature was published within the last few years, indicating a new interest in this area.

This review is not without limitations. First, like many other qualitative research, it is not possible to draw conclusion on causality or generalizability. If this review was substantiated by quantitative component, implications for clinical practice can be made. However, it was beyond the scope of this review. We contend, however, on the credibility of the information, and the transferability of the theoretical knowledge that is applicable to other similar group of women.

Second, although one study included in the review may not met the criteria of high quality research, the expressions of experience offer an insight into important research concepts and questions and contribute to the richness of information. Third, although we have searched multiple databases and the grey literature, we did not include qualitative data in national and regional confidential inquiry reports, unpublished data presented at smaller conferences, or studies published in foreign language and non-indexed journals. Fourth, the majority of the studies were conducted in developing countries, and this review therefore does not represent the complete global view of women with severe maternal morbidity. Finally, more studies are needed to develop further understanding of the differences experienced by the women in various health care systems and communities.

## Conclusion

This metasynthesis reveals three components in the experience of women with severe maternal morbidity that are related to the provision of care, severe maternal morbidity itself and health care seeking behavior. The barriers to the access and utilization of heath care services were identified through the women’s lived experiences and perspectives. Studies on the experience of severe maternal morbidities are few, and the health seeking behavior of women has seldom been documented. Their mental health outcomes were poor and persisted in the first three months after birth. Hence, there is a need to identify the persistence and severity of maternal morbidity, in addition to physical and sexual health, over a longer period of time. Studies conducted within the hospital setting are more prone to information bias as the women may be reluctant to criticize the facility. More realistic information may be obtained in community-based interviews. The impact of negative fetal outcomes would be a strong influencing factor both in the women's coping mechanisms and their perception towards health care. These findings may help to increase awareness regarding the physical and non-physical components of severe maternal morbidity and provide guidance for professionals about various levels of preventive measures.

## Supporting Information

S1 PRISMA Checklist PRISMA 2009 checklist. (DOC)

S1 Fig PRISMA 2009 flow diagram. (DOC)
