# Supplementary material for: Metasynthesis: Experiences of Women with Severe Maternal Morbidity and Their Perception of the Quality of Health Care
Source: PLoS One. 2015 Jul 1;10(7):e0130452. doi: 10.1371/journal.pone.0130452 (PMC4488589; doi:10.1371/journal.pone.0130452)
Supplement: S1 Fig — (DOC) [file pone.0130452.s002.doc]

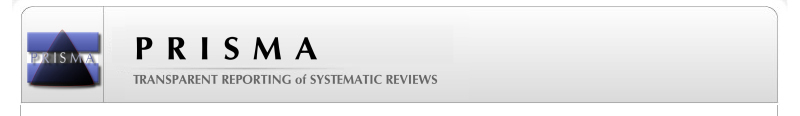
**PRISMA 2009 Flow Diagram**

**Screening**

**Included**

**Eligibility**

**Identification**

Records identified through database searching
(n = )

Additional records identified through other sources
(n = )

Records after duplicates removed
(n = )

Records screened
(n = 276)

Records excluded
(n = 265)

Full-text articles assessed for eligibility
(n = 11 )

Full-text articles excluded, with reasons
(n = 2 - not in English language )

Studies included in qualitative synthesis
(n = 9 )

Studies included in qualitative synthesis (meta-synthesis
(n = 9 )
